# P-1041. A Comparison of Pre- and Post-Pandemic Infection Epidemiology in Patients with Extracorporeal Membrane Oxygenation (ECMO)

**DOI:** 10.1093/ofid/ofaf695.1236

**Published:** 2026-01-11

**Authors:** Shardul N Rathod, Parmeen Singh H Bindra, Heather Byrd, Randy McGregor, Rebecca S Harap, Benjamin Bryner, Kalvin Lung, Kendall Kling

**Affiliations:** Northwestern Memorial Hospital, Chicago, Illinois; Northwestern University, Chicago, Illinois; Northwestern University, Chicago, Illinois; Northwestern Medicine, Chicago, Illinois; Northwestern Memorial Hospital, Chicago, Illinois; Northwestern University Feinberg School of Medicine, Chicago, Illinois; Northwestern University Feinberg School of Medicine, Chicago, Illinois; Northwestern University, Chicago, Illinois

## Abstract

**Background:**

Extracorporeal membrane oxygenation (ECMO) is a mechanical circulatory device that supports patients with pulmonary or cardiac failure. Patients on ECMO have a significant infection risk due to cannula insertion into large vessels with evidence suggesting that 20% of patients develop an infection. The COVID-19 pandemic led to increased ECMO utilization, though it is unclear if ECMO-related infection epidemiology has shifted. We performed a retrospective comparative analysis of infection epidemiology in ECMO patients pre- and post-pandemic.

**Table 1:**
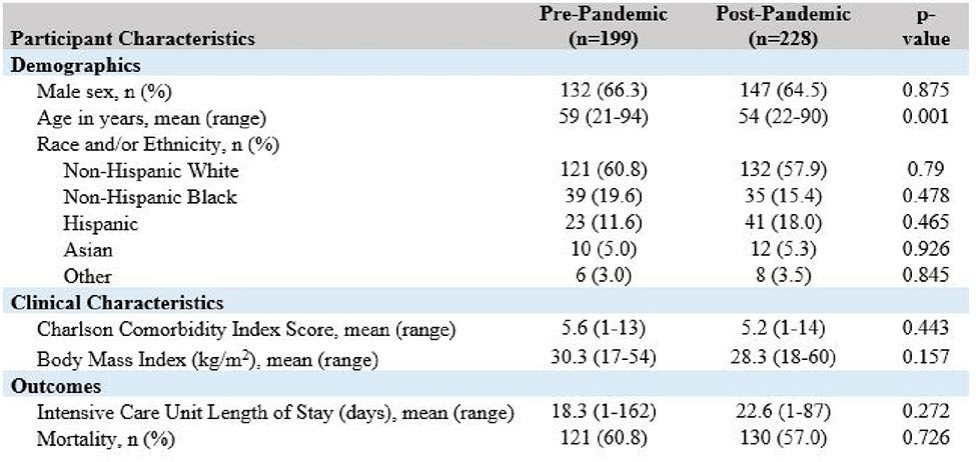
Participant characteristics

**Table 2:**
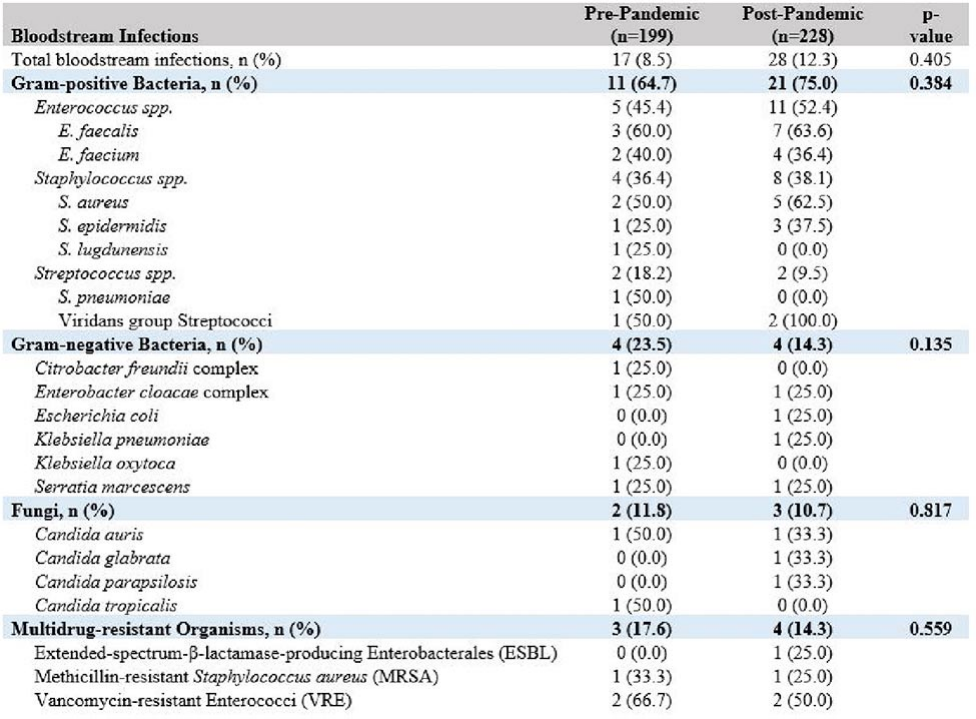
Bloodstream infections during ECMO Abbreviations: ECMO: extracorporeal membrane oxygenation

**Methods:**

After institutional review board (IRB) approval, infection data was collected from the ELSO Registry and our Enterprise Data Warehouse. The pre-pandemic range was defined as 4/1/2018-2/29/2020 and the post-pandemic range was defined as 3/1/2020-2/1/2022.

**Table 3:**
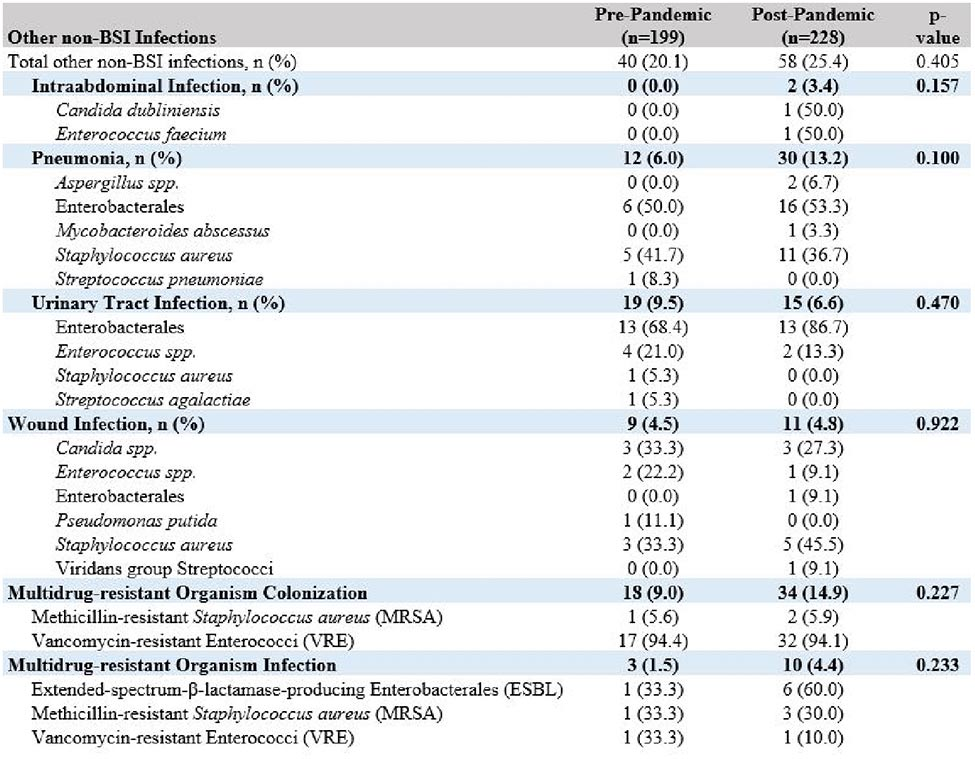
Other Non-BSI Infections during ECMO Abbreviations: BSI: bloodstream infection; ECMO: extracorporeal membrane oxygenation

**Results:**

We assessed 199 patients pre-pandemic and 228 patients post-pandemic. Most patients in both cohorts were male with a mean Charlson score of 5.2-5.6 and mean BMI of 28-30 (Table 1). There were no significant differences in race, ethnicity, ICU length of stay (LOS), or mortality pre- and post-pandemic; patients post-pandemic were younger overall.

Incidence of bloodstream infections (BSIs) was similar in both groups (Table 2). Gram-positive bacteria comprised 65-75% of BSIs, with *E. faecalis* the most common organism in both groups. BSIs caused by Gram-negative bacteria comprised 14-24% of cases in both groups. BSIs caused by *Candida spp*. were observed in 11-12% of cases and most commonly due to *C. auris*. Multidrug-resistant organisms (MDROs) caused 14-18% of BSIs and typically were vancomycin-resistant Enterococci. Other non-BSI infections occurred in 20-25% of patients, and the frequency of non-BSI infections did not differ between groups (Table 3). MDRO colonization or infection did not differ between groups.

**Conclusion:**

ECMO remains an important life-saving procedure for critically ill patients, and infectious complications occur in up to 20% of patients. This study demonstrated that amongst ECMO recipients at our institution, the post-pandemic state led to no differences regarding infection rate, MDRO colonization or infection, mortality, or ICU LOS.

**Disclosures:**

All Authors: No reported disclosures

